# Treatment of Complex Mandibular Body Fractures and Functional Reimplantation of the Maxillary Alveolar Fragment

**DOI:** 10.1155/2020/8853562

**Published:** 2020-09-26

**Authors:** Beatriz Sobrinho Sangalette, Rafaella Ferrari Pavoni, Thayna da Silva Emídio, Tiago Levatti, Marcos Capelari, Cláudio Maldonado Pastori, Gustavo Lopes Toledo

**Affiliations:** ^1^Department of Biological Sciences-Anatomy, Bauru School of Dentistry, University of São Paulo-FOB/USP Alameda Octávio Pinheiro Brisola, n° 9-75, Zip Code, 17012-901 Bauru-SP, Brazil; ^2^State University of North Paraná-UENP. Av. Getúlio Vargas, no. 850, Zip Code, 86400-000 Jacarezinho, PR, Brazil; ^3^Marília University-UNIMAR. Av. Higino Muzi Filho, n° 1001, Zip Code, 17525-902 Marília, SP, Brazil; ^4^Oral Maxillofacial Surgery Department, Santa Casa da Misericórdia de Santa Cruz do Rio Pardo. Av. Dr. Ciro Melo Camarinha, n° 530, Zip Code, 18900-000 Santa Cruz do Rio Pardo, SP, Brazil; ^5^Department of Bucomaxilofacial Surgery, University Center of Adamantina-UNIFAI. Av. Francisco Bellusci, n° 1000, CEP, 17800-000 Adamantina, SP, Brazil; ^6^Oral Maxillofacial Surgery Department, State University of North Paraná-UENP. Av. Getúlio Vargas, no. 850, Zip code, 86400-000 Jacarezinho, PR, Brazil

## Abstract

**Introduction:**

This report aims at demonstrating the treatment of complex mandibular fracture functional reimplantation of the maxillary alveolar fragment (FRAF), denoting the possibility and feasibility of this reduction with an excellent prognosis. *Case Report*. Patient E.M.S, 25 years old, male, leucoderma, referred to the Emergency Room of our institute. He reported being a victim of physical aggression, occlusal alteration, limitation of mouth opening, sensibility loss in the mentalis region, right infraorbital, and denied visual alteration. On physical examination, during the inspection and palpation, the crackling was observed in the right mandibular region and apical displacement of the maxillary alveolar process, corresponding to elements 13, 14, and 15.

**Conclusion:**

The rigid fixation of the complex jaw fracture and alveolar maxilla process, through functional reduction, indicated satisfactory applicability, and favorable prognosis.

## 1. Introduction

The bones of the face are part of the complex stomatognathic apparatus, of which the mandible occupies the second position among the most affected in craniofacial traumas [1]. Such prominence is related to the anatomy and projected anatomical disposition itself in relation to the other bones face, being preceded only by the nasal bones that occupy a prominent position when compared to the mandible [1, 2].

The etiology of mandibular fractures is concentrated in cases of physical aggression and automobile accidents, although sports accidents and pathological or mechanical fractures are present as predisposing factors, either in the involvement of neoplasms or during the extraction of lower third molars [1, 2]. Commonly, there are associated dentoalveolar fractures, varying with the direction and impact of the trauma, involving the alveolar process, adjacent teeth, and soft tissues [3].

For diagnosis, a thorough clinical and radiographic examination should be performed, taking into account factors such as impact energy, direction, and location, as well as the resilience of the structures involved [4]. The objective of this study was to report the treatment of complex mandibular fracture and block fracture of the alveolar process in the maxilla, involving dental elements, denoting singular technique of functional alveolar reimplantation of the respective alveolar bone fragment (FRAF), besides primary stabilization of the mandibular fracture before reconstruction plate placement, thus preserving the structure and restoring function.

## 2. Case Presentation

**Figure 1:**
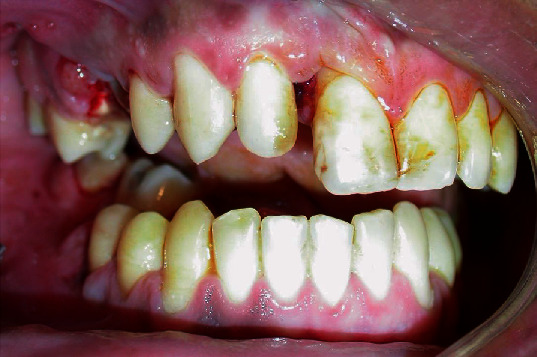
Initial appearance of the intraoperative showing apical fragment intrusion.

Patient E.M.S, 25 years old, male, leucoderma, was referred to the Emergency Room of our institute. During a medical-dental questionnaire, the patient mentioned having been a victim of physical aggression, reporting occlusal alteration, limitation of the amplitude of buccal opening, and loss of sensibility in the mental and infraorbital region. He denied visual and respiratory changes, although there were signs of subconjunctival ecchymosis and epistaxis. On physical examination, during the inspection and palpation, crepitation was observed in the body region and right mandibular angle and apical displacement of the bone fragment in the premaxilla, corresponding to elements 13, 14, and 15, with an evident sign of alveolar tooth fracture (Figure 1). In all maneuvers, the patient reported severe pain and great discomfort. Orthopantomographic radiography showed an image suggestive of loss of bone tissue continuity solution. However, the fragmentation of the alveolar process was obscure, with only sinus veining on the corresponding side, given the blood collection in the cavity.

**Figure 2:**
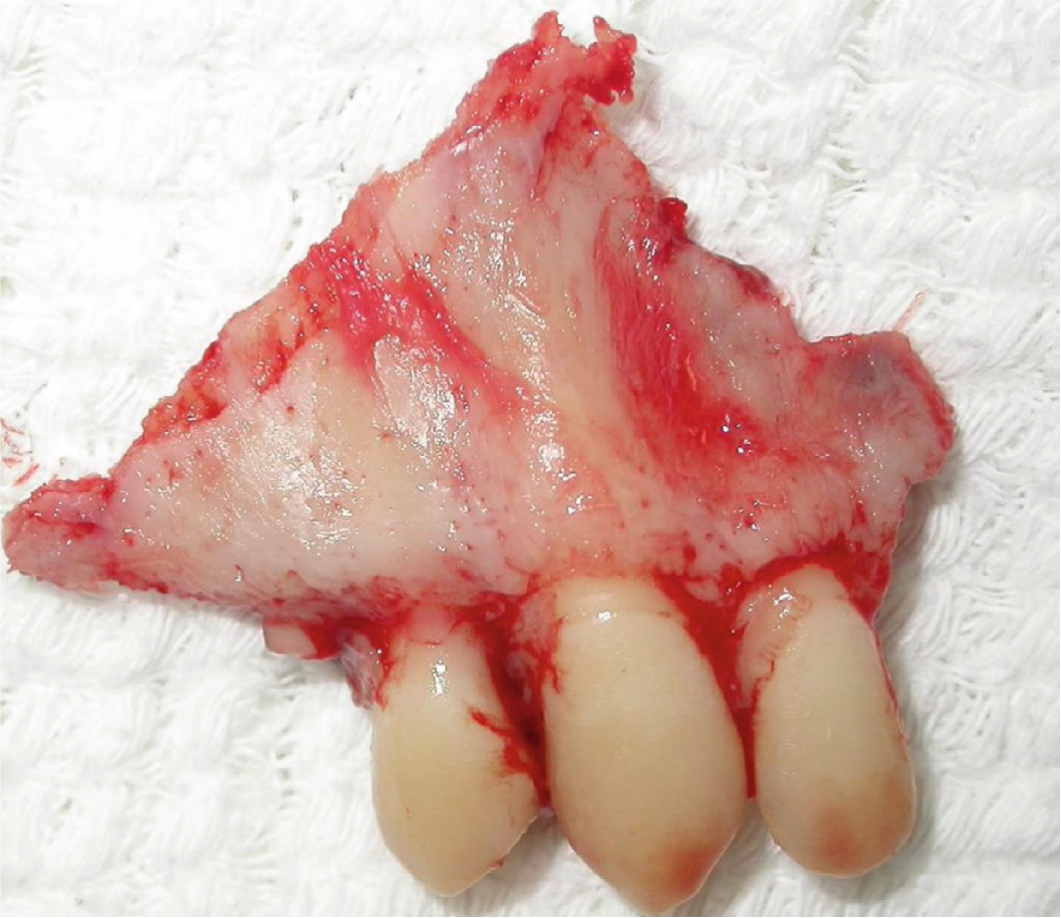
Alveolar fragment after removal of granulation tissue brought between it and the maxilla.

**Figure 3:**
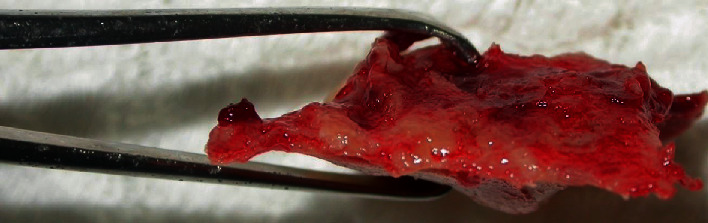
Super-inferior view of the premaxilla alveolar fragment.

**Figure 4:**
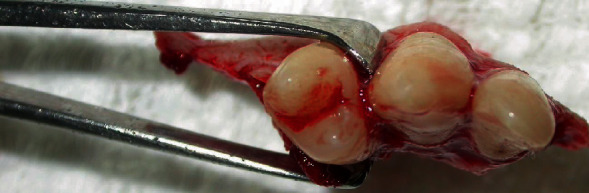
Occlusal view of the premaxilla alveolar fragment.

**Figure 5:**
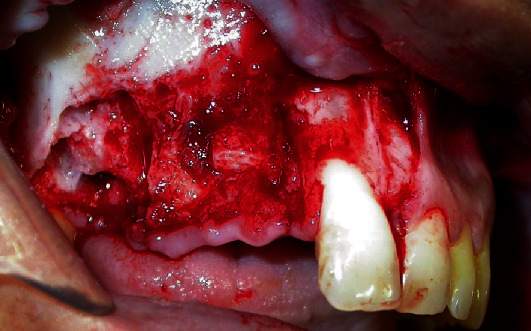
Curved surgical bed for functional reimplantation of the fragment.

**Figure 6:**
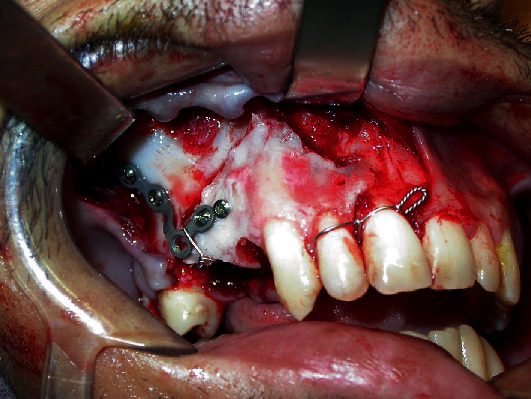
Functional reduction of the fragment with the aid of the “L” plate and 2.0 system titanium screws.

After systemic stabilization, the patient was taken to the Buccomaxillofacial Surgery and Traumatology Service, after 10 days of the first care. In the surgical center, in HDD, under general anesthesia, antisepsis was started with topical PVP-i and placement of the sterile fields, followed by subperiosteal infiltrative terminal anesthesia in the right maxilla region, aiming at facilitating the detachment of the muco-suspicious flap and promoting hemostasis; through the Newman Modified incision, the detachment of the flap continued, evidencing the dentoalveolar fracture. In order to eliminate the granulation tissue at the interface of the fragment and maxilla, which would certainly prevent good bone repair, the fragment was removed (Figures 2–4). However, it was again juxtaposed through the FRAF technique, which consists of the removal of the bone fragment and subsequent repositioning of it in its pretraumatic place, being fixed by using guided plates and titanium screws, by steel wires positioned in Modified Kazagian ties (Figures 5 and 6). Note the absence of the element 15 in Figure 6, extracted due to loss of bone support, is understood as functional reduction when the occlusion prior to trauma is overcome through occlusal wear or even dental impression, which does not necessarily mean Angle Class I.

**Figure 7:**
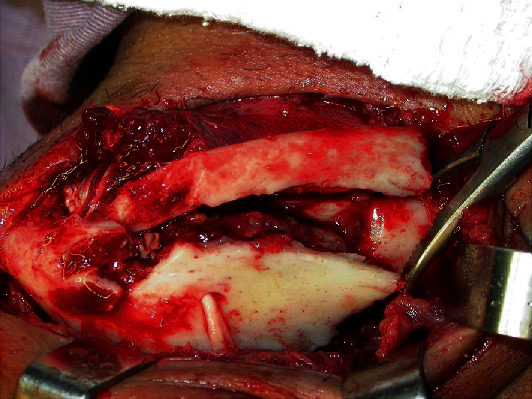
Exposure of complex mandible fracture.

**Figure 8:**
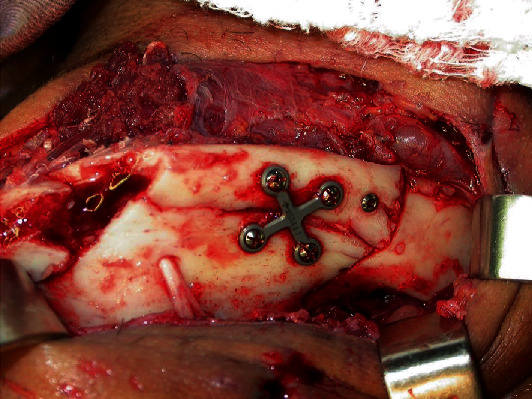
Reduction and stabilization of fractures with the lag screw, X-plate, and 2.0 system titanium screws.

**Figure 9:**
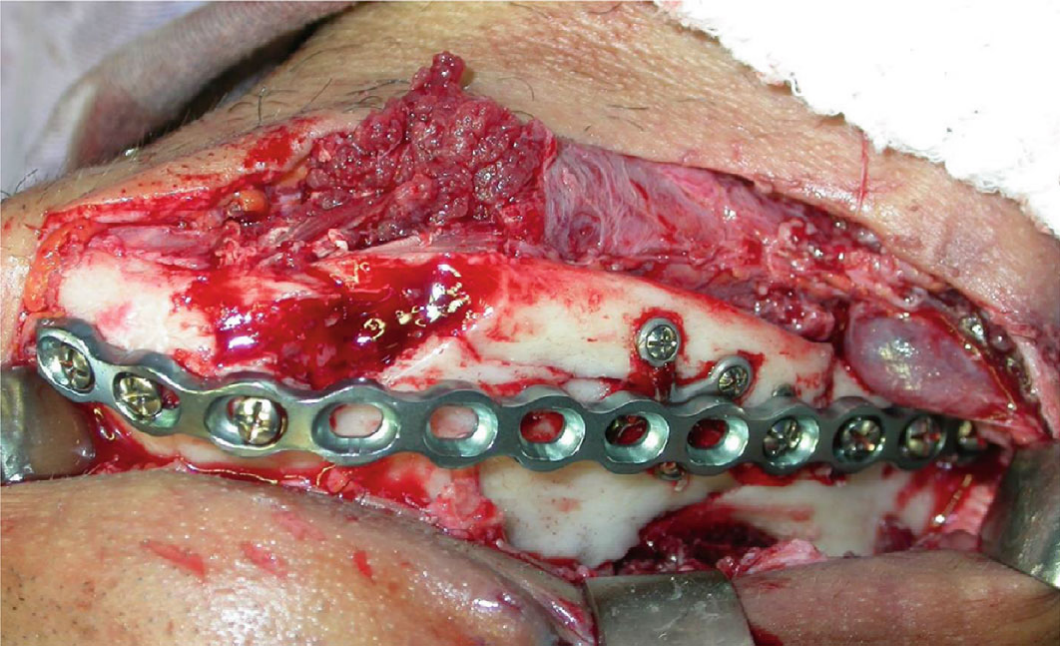
Rebuild plate installation overlapping stabilization material.

In the next step, through the Risdon incision in the mandible, the subcutaneous planes were approached, location and protection of the facial nerve and artery, and incision of the pterygomassetric band and detachment. With the separation of the fragments and evaluation of the lingual space, the reconstruction and anatomical repositioning of the fragments with transfixing screws and plates and screws of the 2.0 system (Figures 7 and 8) were started. To support the reduction, a plaque reconstruction was attempted, which promoted stability and the possibility of functional adaptation to occlusion (Figure 9).

The suture of the deep planes of the mandibular approach and oral cavity was performed with Vicryl 4.0 wire and on the skin was used Nylon 6.0.

The patient was preoperatively treated with Dexamethasone 8 mg, Cephalotin 1 g, and intravenous dipyrone 500 mg. All medications were maintained in trans and postoperative and were adapted for use in tablets. Postoperatively, N-acetylcysteine 500 mg was added to the bucosinusal communication.

### 2.1. Outcome and Follow-Up

**Figure 10:**
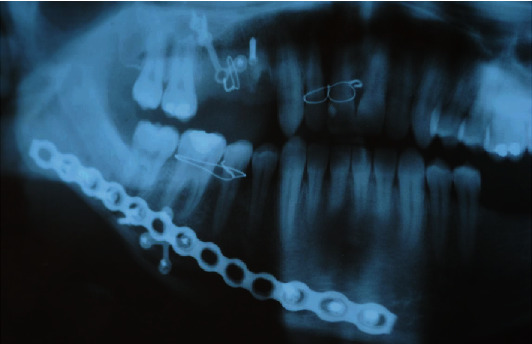
Orthopantomographic radiographic image of titanium plates and screws in position.

**Figure 11:**
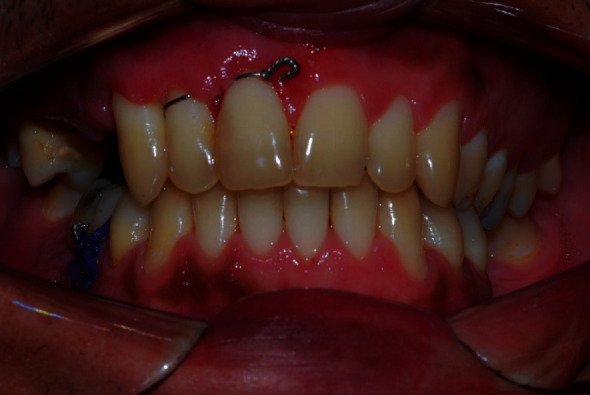
Postoperative appearance of 7 days, good healing aspect of the surgical approach, suture stitches in position, and reestablishment of functional occlusion.

The patient followed postoperative follow-up for 7, 14, 21, 35, and 64 days, presenting evolution within the norms of normality and cicatricial process compatible with the surgical procedure performed. There was an absence of secondary infections, occlusal restoration, and consolidation of the fragment in the maxilla. In respect to the regeneration of the nervous tissue, although it is known, by the literature, that the regeneration of the proximal stump occurs, in this specific case, it comes to an avulsion of fragment with involved teeth, which made the reasoning of this case to devolve in the same conditions and treatment of avulsioned teeth, that is, endodontic treatment after 14 days of the trauma. The patient was referred to the endodontic treatment of the elements 13 and 14, making it possible to maintain them in the oral cavity. A radiographic examination of the control was performed, in which titanium plates and screws were positioned, indicating a satisfactory reduction of the bone fragments (Figures 10 and 11).

## 3. Discussion

Facial fractures generate discussions among students ever since Hippocrates was alive, especially regarding the origin of the trauma, diagnosis, and treatment. In agreement with the findings found in the literature, this report presents a patient who suffered physical aggression of high intensity, generating maxillomandibular trauma. Such an etiology ranks first among the most common causes attended in emergency services [3, 4].

Among the anatomical subdivisions of the mandible, the body region corresponds to a significant percentage of the fractures, and the immediate reduction of the stumps is a determinant factor to obtain adequate occlusion [4, 5]. In the present case, the reduction was mediated due to the need for systemic stabilization of the victim, which has not been shown to cause significant harm to the resolution of the case. Literary scarcity is observed in cases where there is a fragmentation of the mandibular body with mediate reduction, demonstrating the relevance of this report to the scientific area.

The treatment choice was based on the analysis of complete clinical and radiographic examination, considering the possible sequels, both aesthetic and functional, that could be caused. According to most of the cases found in the literature, even in the other mandibular regions, the functional reduction was obtained through occlusal adjustment and rigid internal fixation, considering the complexity and biases of the reduction of complex fractures or maxillo-mandibular fractures [1, 5–7]. What has seemed a more viable solution in patients with great dental loss and inadequate occlusion.

According to a study carried out by Melo and Oleveira (2013), dentoalveolar trauma resulting from physical aggression accounts for about 35.8% of the cases, a study carried out on 1459 patients showed that in only 1% of the cases there was an association of dentoalveolar trauma with mandible fracture [8]. Although there are few cases in the literature, the treatment of dentoalveolar trauma with bone fragment removal and repositioning should be performed, taking into consideration the adjacent structures and stage of dental development when there are elements involved [9]. However, the functional alveolar reimplantation technique advocated by the authors showed favorable results, indicating the viability and success of the technique provided that the reduction and fixation principles presented in the surgical steps have been obeyed.

It is important to observe that the removal of the fragment for removal of granulation tissue was indispensable; its nutrition was safeguarded by the periosteum itself carefully detached in the step of dieresis [10]. From its removal, it served as a free graft, a technique perfectly supported in the literature since it establishes a standard limit of up to 6 cm for its biological viability [11–13]. In addition, it is possible to verify the veracity of the postoperative orientation that did not show any signs of resorption or infectious processes. The fact that the noncoaptation of the fragment to the maxilla bone could be rejected could lead to osteomyelitis, a more damaging and unfavorable prognosis.

The nonrigid attachment of the dental elements involved should be indicated, when necessary, to stabilize them [14–16]. However, in this case, there was a fracture of the alveolar bone plates, where the technique recommended by the care team consisted of the use of rigid fixation of the fragment with titanium plates and screws, since there was enough room to use them.

With the evolution of the case, we can conclude that the proposed treatment proved to be effective, reestablishing the patient functionally. The FRAF technique process and primary stabilization of the anterior mandibular complex fracture and reconstruction plate are feasible and demonstrate a favorable prognosis when correctly indicated, following the basic principles of reduction, stabilization, and fixation even if mediated.
